# Recent Developments at DG Competition: 2016/2017

**DOI:** 10.1007/s11151-017-9592-x

**Published:** 2017-11-09

**Authors:** Benno Buehler, Daniel Coublucq, Cyril Hariton, Gregor Langus, Tommaso Valletti

**Affiliations:** 1 Directorate-General for Competition, European Commission, MADO 17/051, 1049 Brussels, Belgium; 2 Directorate-General for Competition, European Commission, MADO 17/048, 1049 Brussels, Belgium; 3 Directorate-General for Competition, European Commission, MADO 17/37, 1049 Brussels, Belgium; 4 Directorate-General for Competition, European Commission, MADO 17/010, 1049 Brussels, Belgium; 5 Directorate-General for Competition, European Commission, MADO 17/026, 1049 Brussels, Belgium; 6 Imperial College Business School, South Kensington Campus, London, SW7 2AZ UK

**Keywords:** Antitrust, Competition policy, Innovation, Mergers, Most favoured nation clause, State aid

## Abstract

The Directorate General for Competition at the European Commission enforces competition law in the areas of antitrust, merger control, and state aids. This year’s article provides first a general presentation of the role of the Chief Competition Economist’s team and surveys the main achievements of the Directorate General for Competition over 2016/2017. The article then reviews the economic work undertaken in one merger case between Dow/DuPont, which raised specific issues related to innovation, as well as in an antitrust case on parity clauses related to Amazon e-books.

## Main Developments in 2016/2017

### The Chief Competition Economist Team

The Chief Competition Economist’s team (“CET”) is a part of the Directorate General for Competition of the European Commission (“DG Comp”), which was created in 2003 to assist in evaluating the economic impact of its actions. The CET’s staff consists of 30 economists (mostly holding Ph.D.s) with a mix of permanent and temporary positions. The head of the CET—the Chief Competition Economist—is recruited outside the European Commission and has a three-year term.

The CET has both a support role and a scrutiny role. As part of its support role, it provides guidance on methodological issues of economics and econometrics in the application of EU competition rules. It contributes to individual competition cases—in particular, the ones that involve complex economic issues and quantitative analysis—and to the development of general policy instruments, as well as assisting with cases that are pending before the Community Courts. Members that are seconded from the CET to case teams have a specific and independent status and report directly to the Chief Competition Economist. As part of the scrutiny role, the Chief Competition Economist can report his opinion directly to the Director-General of DG Comp as well as to the Competition Commissioner, providing her with an independent opinion on the economic aspects of a case before she proposes a final decision to the European Commission.

The CET is active in DG Comp’s three main areas of policy areas: antitrust, mergers, and acquisitions, and state aid. Historically, the CET’s main domain of activity is merger investigations—typically 50–60% of CET’s time—while state aid and antitrust each takes roughly 20–25% of CET’s work allocation.

### DG Competition’s Activities in 2016/2017

#### Antitrust

Between January 2016 and July 2017, the Commission took decisions in six (non-cartel) antitrust cases: Container Shipping,^1^ CDS—Information market,^2^ and Cross-border access to pay-TV (Paramount),^3^ all three of which were in July 2016; ARA foreclosure^4^ in September 2016; E-book MFNs and related matters (Amazon)^5^ in May 2017; and Google Search (Shopping)^6^ in June 2017. A short description of the Google Search case is given immediately below, while Sect. 3 provides details of the CET’s work in the Amazon e-book MFN case.

#### The Google Search (Shopping) Case

In the Google Search (Shopping) case, the European Commission found that Google had abused its market dominance as a search engine throughout the European Economic Area by giving an illegal advantage to another Google product: its comparison shopping service. While this case is not further developed in this article, it has attracted considerable attention.

In a nutshell, according to the Commission’s findings, Google had systematically given prominent placement to its own comparison shopping service, which were placed above the results that Google’s generic search algorithms consider most relevant whenever a consumer typed a product-related query into the Google general search engine. By contrast, rival comparison shopping services were subject to Google’s generic search algorithms, including demotions (which lower a search entry’s rank in Google’s search results).

The European Commission decision did not object to the design of Google’s generic search algorithms or to demotions as such, nor to the way that Google displayed or organised its search results pages; but the Commission objected to the fact that Google had leveraged its market dominance in general internet search into a separate market—comparison shopping—in order to promote its own comparison shopping service in search results, whilst demoting those of rivals. In practice, this meant that consumers very rarely saw rival comparison shopping services in Google’s search results.

According to the Commission’s decision, this conduct had a significant impact on competition in comparison shopping markets, as consumers generally clicked far more on search results at or near the top of the first search-results page than on results lower down the first page, or on subsequent pages, where rival comparison shopping services were most often found after demotion. Since the start of the abuse in each country of the European Economic Area, Google’s comparison shopping service made significant gains in traffic, whilst rival comparison shopping services suffered a decrease in traffic from Google’s search results pages on a lasting basis.

One aspect of the case in which the CET took an active role was the assessment of experiments that were run by Google on its search interface. Using data that were generated by changes in the display of Google’s Shopping Unit, the Commission evaluated the impact on the traffic of the competitors of Google’s shopping service. The Commission concluded that the display of the Shopping Unit that the Commission identified as favourable to Google, did indeed have significant negative consequences on traffic of the competitors.

In order to comply with the decision, Google announced on 28 September 2017 that it would open the Shopping Unit to the competitors of Google’s shopping service. Competitors are allowed to participate in an auction for display slots in the Shopping Unit, similarly to Google’s own shopping service. Google’s shopping service is to be operated under separate accounts and has to be financially self-sustaining in order to assure that it does not receive privileged treatment from its parent company. The remedy is subject to the Commission’s evaluation that also involves an external expert.

#### Mergers

During the period January 2016 to July 2017, 590 merger transactions were notified to DG Comp.^7^ A significant share of these cases (412, i.e. 70%) are so-called “simplified” cases that require a simple assessment in which the CET is not involved. The remainder of the cases are subject to a “first-phase” investigation of 25 working days, which typically ends in a clearance Decision without remedies (128 cases) or with remedies (33 cases).

When remedies at the end of the first-phase investigation are not adequate to resolve the competitive concerns identified at that stage, or if none are submitted, the transaction is subject to a “second-phase” or “in-depth” investigation, which lasts 90 working days.^8^ A major step of this phase is the issuance of a Statement of Objections, which informs the Parties to the transaction of the European Commission’s preliminary conclusions. During this period, the majority of the second-phase cases were authorized subject to remedies (eight cases), whilst three case were prohibited, and one was withdrawn after the submissions of the Statement of Objections.^9^

The CET is typically involved in all second-phase investigations and very often involved in complex first-phase investigations.

#### Innovation Concerns in Mergers

Most assessments of merger transactions are related to unilateral effects on price. Nevertheless, competition authorities have long been concerned with the potential effects of mergers on innovation. The European Commission Horizontal Merger Guidelines mention that effective competition benefits consumers by promoting innovation and that mergers may deprive consumers of the benefit of improved and new products.^10^,^11^ Since 2014, theories of harm that are based on innovation concerns have been pursued by DG Comp^12^ in at least seven merger cases and upheld by the Court of Justice of the European Union in another case.^13^

Innovation concerns normally emerge in markets or industries in which the merging parties have strong innovation capabilities and/or track records, are facing few rivals with such capabilities and where barriers to entry in research and development (“R&D”) are significant. The innovation concerns are related to the possibility that, following the transaction: (1) the merging parties will limit future price competition between their forthcoming and their existing products, or between their forthcoming products; and (2) the merged entity will discontinue, delay, or redirect its efforts in each of the merging parties’ pre-merger lines of research. The innovation concerns are also related to the possibility that: (3) the transaction will remove one of the few competitors with innovation capabilities in these markets.

In the pharmaceutical and agrochemical industries, the Commission typically assessed overlaps between the merging firms’ pipelines (at various stages of their development) as well as with their current products, and the overlap between their lines of research.^14^ In engineering sectors—in particular in industrial gas turbines and in oil-field products—the Commission reviewed the R&D capabilities of the merging parties and their main competitors, usually in terms of R&D strength and track record, in particular with a view of ensuring that remedies replicate the competitive constraints that would be lost as a result of the merger.^15^ In services areas, such as financial exchanges, the Commission analysed innovations related to products, technology, process, and market design.

Section 2 below provides details of the CET’s work in the Dow/DuPont case, which hinge upon, in particular, concerns that are related to innovation.

#### State Aid

During the period 2016–2017, state aid control has been characterized by enforcement under the new state aid architecture following the State Aid Modernization initiative (SAM). The SAM has led to a significant decrease in the number of notifications, as more state aid is covered by the General Block Exemption Regulation (GBER), which enables Member States to grant subsidies that satisfy a number of conditions without prior approval from the Commission. This enables the Commission to focus more resources on assessing state measures that are a priori potentially more distortionary.

In 2016, the Commission took 535 decisions in the area of state aid, most of which were compatible or no aid decisions.^16^ An important area of activity concerned tax rulings, with the adoption of the Apple recovery decision^17^ and the opening of a new investigation of possible aid to GDF Suez (Engie) in Luxembourg.^18^ The Commission has continued to approve a number of ex-post evaluation plans that cover many areas of government spending, such as R&D tax credits, regional aid, subsidies for renewable energy, subsidies for broadband investment, etc. In a large number of these plans, Member States have committed to implement evaluation techniques (such as regression discontinuity design) in line with the Staff Working Document that was published by the Commission in 2013.^19^ As a follow-up to the capacity mechanisms sector enquiry,^20^ the Commission is also assessing the designs of a large number of individual capacity mechanisms across Member States.

## Dow/DuPont merger

### Background

On 27 March 2017 the Commission cleared a merger between Dow and DuPont, subject to conditions.^21^,^22^ Dow and DuPont were active in particular in the discovery, development and formulation and sales of crop-protection products. The markets for crop-protection products are highly concentrated and are characterized by high barriers to entry.

The Commission identified competition concerns based on overlaps between existing-to-existing products, forthcoming-to-forthcoming products in development stages, and forthcoming-to-existing products, notably in selective herbicides and insecticides.^23^ The Commission also found that the Parties had similar research capabilities and a number of overlapping lines of research for new active ingredients (“AIs”) in the discovery stage.^24^ The Commission investigated the extent to which the transaction would decrease innovation incentives of the merged firm and the associated risk of discontinuation, delay, or redirection of existing overlapping lines of research, as well as the risk that the merged firm would reduce its discovery efforts in relation to new AIs.

The next two sections focus on the main contributions of the CET to the Commission’s competitive assessment of this case; both were related to the effect of the merger on innovation competition.

### Economic Framework

The economic literature indicates that a merger between rival innovators can affect the incentives of the merged entity to innovate via three main channels: (a) innovation rivalry; (b) price coordination; and (c) efficiencies.

A merger can, foremost, reduce innovation incentives by suppressing innovation competition between the merging parties: the innovation rivalry channel. Prior to the merger, the merging parties would have an incentive to capture profitable sales from each other by introducing new and improved products. The merger removes this incentive, which depresses innovation by the merging firms. This effect can be thought of as a standard unilateral merger effect, applied to innovation effort rather than to prices or volumes (as in a conventional/static unilateral effects analysis). Similar to the standard unilateral price effect, the effect of a loss of innovation competition can be significant when: (1) the merger brings together two important innovators out of a limited number of innovators; and (2) the merging parties absent the merger would have been likely to divert to a significant extent future sales from each other by investing in innovation (i.e., the merging parties are close innovation competitors).^25^

The innovation rivalry channel has been analysed in the economic literature: Farrell and Shapiro (2010), for example, introduce the closely related notion of the “innovation diversion ratio”. This concept has been subsequently emphasized in some policy works: e.g., Shapiro (2012) and Gilbert and Greene (2015). The innovation diversion effect is also formally captured in models of cooperation in cost-reducing R&D.^26^

The second channel for merger effects on innovation relates to the elimination of future product market competition between the merging parties. Following a merger, the merged firm will coordinate the pricing of its future competing products to increase its overall profits: the price coordination channel. A less intense competition in the product market increases the profits from innovating (profits from a successful innovation), which boosts innovation incentives. The less intense competition, however, also tends to increase the profits from not innovating and so depresses innovation incentives. Therefore, in contrast to the loss of innovation rivalry, less intense competition in the product market following a merger has in principle an ambiguous effect on the incentive of the merging firms to innovate.

A number of papers in the economic literature analyse the impact of changes of product market competition on innovation in isolation from innovation rivalry, with mixed findings.^27^ While some papers find that greater product market competition spurs innovation—e.g., Arrow (1962)—others find the reverse under some conditions; e.g., Aghion et al. (2005) propose that the overall relationship between competition and product market competition has an inverted-U shape. None of these papers studies directly the impact of mergers on innovation; instead, they refer to competition more loosely. It is therefore difficult to draw implications for merger control from this strand of the literature.^28^

In a merger, the two effects—the loss of innovation rivalry and the loss of future product market competition—act on innovation incentives of the merging firms simultaneously and possibly in different directions. Moreover, the non-merging firms may increase innovation in response to any reduction in innovation by the merging firms, which would reduce any negative impact of the merger on the overall industry innovation. Recent economic literature thus employs economic models to analyse the likely effects of a merger on innovation via the two channels simultaneously, while taking into account the reaction of non-merging firms. This literature finds that the merged firm reduce innovation compared to premerger levels and that any response by non-merging rivals tends to be insufficient to compensate for that loss of innovation (absent efficiencies).^29^ These findings are consistent with the position taken by a number of policy surveys—e.g., Gilbert (2006), Gilbert and Greene (2015), and Shapiro (2012)—that a merger between two out of a few rival innovators is likely to reduce innovation incentives in the absence of merger-related efficiencies.

A merger may also generate efficiencies that favour innovation. In Dow/DuPont, the Commission considered in particular the notion of appropriability: the ability by an innovator to prevent knowledge spillovers to other firms. If the protection of intellectual property rights (“IPRs”) is weak, a merger may reduce the risk of imitation by a competitor, thus increasing the ability of innovators to appropriate the rewards from innovation efforts. However, this factor is unlikely to play a role when imitation concerns are properly dealt with by effective IPRs.^30^

In Dow/DuPont, the Commission found that potential efficiencies were unlikely to play a significant role, given that innovation in the crop-protection industry mostly takes the form of product innovation that is protected by effective IPR. In other words, appropriability was already high in the absence of the merger. Indeed, most of the innovation in the crop-protection industry takes place via the introduction of new products (i.e. new AIs) that are protected by effective IPR (i.e., patents that are protected for 20 years generally), generating in successful instances significant sales with very high margins both during the patent period and after the patent expires.

Overall, the economic literature shows that closeness and significance of competition between the merging parties are at the core of the analysis of the likely impact of a merger on innovation. The harm to innovation, in the absence of efficiencies, is more likely when: the merging parties have overlapping research targets; the parties are significant competitors in a concentrated industry; and the barriers to entry are high.

### Evidence

#### Qualitative Evidence

The investigation showed that the markets for crop-protection products are highly concentrated. Only five global R&D companies had all of the necessary capabilities to innovate on a global basis: BASF, Bayer, Dow, DuPont, and Syngenta.^31^ Moreover, the barriers to entry in the industry are high. The essential component of a formulated crop protection product is the active ingredient, which is the result of innovation efforts made by R&D agrochemical companies over a period of approximately 10–11 years.

To launch new AIs, crop-protection companies need a complex R&D organisation and specific assets, equipped to: discover new AIs^32^; optimise the molecules (capabilities at the late discovery stage and development capabilities)^33^; and perform the necessary field tests and studies that are required to obtain the approval of a new AI in different world regions. The Commission noted that the R&D costs to develop a new product are significant (USD 280 millions) and have increased sharply over time; the largest increases are related to regulatory changes, which require further regulatory capabilities.

The Commission analysed the Parties’ internal documents to identify the characteristics of research targets, R&D capabilities, and pipelines at the discovery stage (i.e., research stage). The investigation showed that the merging parties had: (1) some overlapping R&D capabilities; (2) some overlapping lines of research in the discovery stage, where the Parties had a number of promising competitive pipeline products; and (3) other overlaps across the different stages of a product lifetime (e.g. discovery-to-development pipelines overlaps and discovery-to-existing product overlaps).^34^,^35^,^36^ Moreover, the Commission found direct evidence on a planned reduction of R&D capabilities compared to the situation without the merger.

The investigation also showed that the remaining three global R&D companies were less effective innovators than the parties for some combinations of crops and pests, due to differentiated innovation assets and capabilities. For these crop-pest combinations, the Commission therefore considered that these competitors were particularly unlikely to offset the risk of a significant loss of innovation competition between the merging parties. The role of other companies that were active in some stages of the innovation process, and most notably Japanese agrochemical companies, is discussed below.

#### Quantitative Evidence from the Analysis of Patent Data

The Commission also carried out an analysis of patent data, which confirmed: (1) the high importance of both merging parties, and in particular one merging party, as innovators; (2) the high degree of concentration in research for new AIs (discovery stage); (3) the significant combined share of research for new AIs accounted by the merging parties, notably in selective herbicides and insecticides; and (4) the closeness between the merging parties in term of innovation efforts.

##### Description of the Data

The Parties provided the Commission with internal databases on patents that were related to crop protection. For each patent, the data contained information on: the publication number; the publication date; whether the patent is related to the discovery stage; and the discipline (i.e., selective herbicides, insecticides, or fungicides). In its analysis, the Commission considered patents filed in Europe with a publication date during the period 2000–2015.

In addition to patents that are owned by the five global R&D companies, a significant number of patents were also owned by other chemical companies, in particular from Japan. In its analysis, the Commission concluded, on the basis of its investigation, that it was not appropriate to treat Japanese companies in the same way as the five global R&D companies: First, their discoveries generally had a limited relevance for the European market.^37^ Second, the few Japanese discoveries that were brought to the European market were mainly done through collaborations/licensing with the five global R&D companies. In other words, the discoveries of Japanese R&D companies can be thought of as an upstream input in the overall R&D activities of the five global integrated players. On that basis, the Commission gave more weight to patent shares that excluded Japanese companies.^38^

In order to assess the parties’ technological importance, the Commission evaluated the quality of the patents that they provided. It is a common practice in the economic literature to use, for this purpose, citation-based measures: based on the number of citations that are accumulated from subsequent patents.^39^ The Commission thus performed such an analysis, using PatentSight’s web-interface^40^ to collect numerous pieces of information on patents, such as their patent family^41^ and the number of citations that were accumulated by a patent family, as well as some other quality measures.^42^

Before presenting the results from the analysis of patent data, Sect. 2.3.2.2 below describes the main methodological issues that were raised by this analysis.^43^

##### Methodological Challenges

First, consistent with the economic literature, the Commission’s analysis showed that patents differ greatly in their technical quality and economic significance: Most patent families are never or rarely cited, and therefore have little technical quality and economic significance, while a few patents account for most of the citations. Given the significant heterogeneity in patent quality, the Commission considered that firms’ technological strength would not be well reflected in simple patent counts per firm and that a citation-based index was more appropriate.

Second, citations of a given patent come from subsequent patents, either owned by the same firm as the one holding the cited patent (referred to as internal citations or self-citations) or owned by different firms (referred to as external citations). The question is then whether internal citations should be given the same weight as external ones in the indices of the firms’ technological strength. The economic literature provides mixed findings in that regard. In particular, while it recognises the importance of internal citations, for example to capture continuous innovation effort in a certain area, it also shows that the relevance of internal citations declines with the size of the patent portfolio that is owned by the firm.^44^ Internal citations can increase automatically with the size of the patent portfolio, regardless of whether these internal citations are indicative of patent quality.

The Commission therefore decided to give more weight to external citations on the basis of the following case-specific facts: One of the merging parties had a very different patent strategy than its competitors, which resulted in a much smaller patent portfolio with significantly higher quality than its competitors.^45^ Moreover, one non-merging party had a patent portfolio of a much greater size than its competitors. The inclusion of internal citations (i.e. using total citations) would thus tend to underestimate the strength of this merging party and risk overestimating the strength of this specific competitor, irrespective of the quality of their respective patent portfolio.^46^

Last, summarizing the quality of each competitor’s patent portfolio in one index requires aggregating the quality of each of its patent families. Given the significant heterogeneity in patent quality, with numerous patents not being cited, the Commission first computed the quality of a patent portfolio as the simple average of the quality of a subsample of the patent families that belonged to this portfolio. The Commission considered various subsamples: in particular, patent families that were above the median quality (referred to as the “top 50% sample” in the Decision), and patent families with quality that was above the 75th percentile (“top 25% sample”).^47^,^48^ Such an approach, which does not consider all patents in the distribution, finds support in the economic literature, in particular Hall et al. (2005).^49^

Alternatively, the literature also provides indication that non-linear weights could be applied to citations to measure the value of a patent portfolio.^50^ Tratjenberg (1990) suggests applying two non-linear weights to the full sample of patents: (1) a 1.1 exponential non-linear weight applied to the number of citations; and (2) a 1.3 exponential non-linear weight applied to the number of citations, which gives more importance to the highly cited patents and therefore to breakthrough innovations. The Commission used this methodology to check the robustness of its quality measures over subsamples. The results that were obtained by applying a 1.1 non-linear weight to the number of citations over the entire sample of patents were very similar to the results obtained with a simple average of the number of citations over the top 50% sample of patents. Moreover, the results obtained by applying a 1.3 non-linear weight to the number of citations over the entire sample of patents were also very similar to the results obtained with a simple average of the number of citations over the top 25% sample of patents.

##### Overview of the Results

First, the patent analysis indicated that both Dow and DuPont were particularly strong innovators on the basis of external as well as total citations. For one merging party in particular, the patent analysis revealed that, while having the smallest patent portfolio, its patents were on average of a significantly higher quality than its competitors; and, as a result, it was either the number-one or number-two innovator for overall crop protection, depending on the measure that was used for patent quality.

Second, Dow and DuPont had a significant combined patent share for the period 2000–2015 for the discovery of new AIs for crop protection, for selective herbicides, and for insecticides, which demonstrated their importance as innovators (both on the basis of external as well as total citations).

Third, the patent shares were also used to calculate concentration indexes (e.g. HHI) for research at the discovery stage (for crop protection overall, for selective herbicides, for insecticides, and for fungicides). This analysis showed a high level of concentration already pre-merger, with a further significant increase in concentration due to the merger, notably for crop protection overall, selective herbicides, and insecticides.

Last, the analysis of the patent data was also helpful to assess the degree of innovation closeness between the merging parties. Indeed, the assessment on innovation closeness described above (see Sect. 2.3.1) relies mainly on current lines of research, based on the review of parties’ internal documents. In order to perform a similar exercise on past lines of research, the Commission identified the best-quality patents of the merging parties and requested the related merging parties’ documents (e.g. presentations, NPV calculations). This analysis revealed that the merging parties have also been close competitors for past innovations.^51^ Overall, the investigation showed that closeness in innovation was persistent, with the merging parties being close innovation competitors both for past innovations and for current innovations.

### Remedy

To address the Commission’s concerns both on product competition and innovation competition, Dow and DuPont offered to divest a large part of DuPont’s herbicide and insecticide businesses, as well as DuPont’s global R&D organisation (including pipelines at the discovery stages for herbicides, insecticides, fungicides, R&D facilities and employees, with the exception of a few limited assets to support the retained business); this would allowing the buyer of the divested businesses to become a global and integrated R&D competitor.

Beyond the elimination of horizontal product overlaps, the divestiture of DuPont’s global R&D organisation not only addressed the Commission’s concerns in innovation competition, but also ensures that the purchaser of the divested businesses would have the capabilities to preserve the viability and competitiveness of the divested current products on a lasting basis throughout their lifecycle.

The divestment package was bought by FMC, a US-based chemicals company. This transaction was approved by the Commission, under conditions, on 27 July 2017.^52^

## The Amazon e-Book Antitrust Case on Parity Clauses

### Brief Description of the Case

The Commission opened proceedings against Amazon in June 2015 in relation to the potential anti-competitive effects of a number of “most favoured nation” clauses (“MFNs”) (also called parity clauses) in Amazon’s agreements with publishers for the distribution of English and German language e-books in the European Economic Area (EEA).

The Commission’s concern was that these clauses—both individually and in combination—may hinder innovation and make it more difficult for competing e-book distributors to differentiate themselves from, and thus compete effectively with, the dominant undertaking Amazon; this would likely lead to anti-competitive foreclosure. The Commission formulated its concerns in a Preliminary Assessment that was issued on 9 December 2016, and the Commission issued on 4 May 2017 a formal commitments decision (“Amazon Decision”).^53^

### Background: Earlier e-Books Case

The Commission had already intervened in the e-book sector in 2012 and 2013 in an earlier antirust case. As will become clear, the earlier e-books case also affected the present case.

In December 2011, the Commission opened proceedings against Apple and five major international publishers: Hachette Livre, Harper Collins, Simon & Schuster, Penguin, and Verlagsgruppe Georg von Holtzbrinck (collectively the “Five Publishers”). The Commission took the preliminary view that the Five Publishers and Apple had engaged in contracts that were aimed at *inter alia* raising the retail prices of e-books by jointly switching the sale of e-books from a wholesale model (where the e-book retailer determines retail prices) to an agency model (where the publisher determines retail prices and the e-book retailer acts merely as its agent) on a global basis, including on Amazon.

In order to eliminate the Commission’s preliminary concerns, the Five Publishers and Apple offered commitments, which were made binding by Commission Decisions in 2012 and 2013.^54^ The commitments included the termination of all relevant agreements and a five-year ban on retail price, wholesale price, and commission parity clauses, as well as a two-year “cooling-off” period whereby the Five Publishers had to allow all e-book retailers that were on agency terms to discount the retail price of e-books up to a level of their respective aggregated annual commissions.

Following the switch by the Five Publishers to the agency model in 2010, an increasing number of publishers followed suit and entered into agency agreements with Amazon.

### Description of the Relevant Clauses in the Amazon MFN Case

For brevity, this article focuses only on a subset of the clauses that were covered by the investigation and the commitments. The following list provides a brief and stylized description of the parity and notification provisions that are relevant for this article.

#### Retail Price Parity Clauses

The Agency Price Parity Clause contractually obliges the e-book supplier (usually a publisher) to set for a given e-book an agency price on Amazon that is not higher than the retail price (including promotions and discounts) at any competing e-book retailer.^55^

The Discount Pool Provision is a term in agency agreements that relates to a “pool” of credits that Amazon may use at its discretion to discount agency prices on Amazon. This pool is filled if the agency price of a given e-book on Amazon exceeds the price of this e-book at competing e-book retailers.

#### Non-price Parity Clauses

The Business Model Parity Clauses contractually oblige the e-book supplier to notify and offer to Amazon the terms for the distribution of e-books under a given business model if that e-book supplier distributes e-books under that business model (for example, reseller, subscription, rental, bundling with physical books or book clubs, by download, partial downloads, streaming or any other form of digital distribution) through any e-book retailer other than Amazon.

The Selection Parity Clauses encompass a number of provisions that impose equal treatment for Amazon with regard to: (1) the catalogue available; (2) the specific format, including its features and functionalities, in which the titles are offered; and (3) the release date of the titles.

#### Retail Price Notification Provisions

These are provisions that contractually oblige the e-book supplier to notify Amazon if the agency price (including promotions) that is set by the e-book supplier on Amazon is higher than the retail price at a competing e-book retailer.

### Preliminary Assessment

The Commission came to the preliminary view that Amazon had abused its dominant position by among others including the Agency Price Parity Clause, the Discount Pool Provision, the Retail Price Notification Provisions (together with requesting price parity), the Selection Parity Clauses and the Business Model Parity Clauses into its agency contracts for the following reasons.^56^

#### Retail Price Parity Clauses

The Commission considered that the Agency Price Parity Clause and the Discount Pool Provision have very similar effects because each of these clauses allows Amazon to ensure that the effective retail price on Amazon does not exceed the retail price of competing e-book retailers.^57^ Therefore, the effect of these Retail Price Parity Clauses is assessed together.

In line with Boik and Corts (2016), the Commission found that the Retail Price Parity Clauses are likely to: (1) deter the expansion or entry of competing e-book retailers, which would strengthen Amazon’s dominant position; and (2) soften competition at the e-books retail distribution level. These two effects could inter alia lead to higher retail e-book prices to the detriment of consumers.

With regard to the first effect, a potential entrant or a competing e-book retailer could in the absence of the Retail Price Parity Clauses attempt to increase its market share by charging a lower commission to e-book suppliers, so as to induce them to set lower retail prices and, hence, attract buyers.

However, if Amazon had agreed on a Retail Price Parity Clause with a given e-book supplier, that e-book supplier could not set lower retail prices (compared to those on Amazon) at any competing e-book retailer. This automatically limited the ability of a competing e-book retailer to attract buyers by offering lower retail prices than those on Amazon. This may have discouraged competing e-book retailers from entering in the first place.

By restricting the scope for offering e-books at competing e-book retailers that were cheaper than on Amazon, price discounts could not be used to compensate customers for switching costs and to induce them to switch away from Amazon. This stifles the entry and expansion of competing e-book retailers in the first place.

A number of market characteristics may have exacerbated this effect. Those include: Amazon’s particularly strong market position; indications that e-book suppliers would support the expansion of competing e-book retailers in the absence of the Retail Price Parity Clauses; and that price appeared to be the most important parameter of competition especially for e-books without sophisticated illustrations or special functionalities.

As regards the second effect, the Commission considered that the Retail Price Parity Clauses are likely to soften competition between e-book retailers that work on the basis of an agency relationship by reducing their incentive to compete on commission fees.

In the absence of the Retail Price Parity Clauses, e-book retailers on agency terms had an incentive to compete on commission fees. Since commissions reduced the profits of e-book suppliers, the latter generally had an interest to steer sales to e-book retailers that charge lower commission fees: for example, by setting lower retail prices on their platforms. For this reason, e-book retailers anticipated that charging lower commission may increase the volumes of e-books that are sold on their platform and thereby increase their overall revenues and profits.

In contrast, where a Retail Price Parity Clause was in place, e-book retailers’ incentives to compete on commission were limited. When charging a lower rate of commission, a competing e-book retailer could no longer expect that e-book suppliers would respond by lowering selectively the retail prices of its e-books on that e-book retailer’s platform. This is because when bound by the Retail Price Parity Clauses, a lower retail price that was set by an e-book supplier on one e-book retailer’s platform would have needed to be matched by an equally low price on Amazon. Hence, a competing e-book retailer could no longer expect to attract customers from Amazon by offering lower rates of commission to e-book suppliers.

Conversely, with a Retail Price Parity Clause in place, as a consequence of a higher commission, e-book suppliers had to raise the retail price of their e-books uniformly on all e-book retailers’ platforms, instead of raising the retail price exclusively on Amazon. Therefore, Amazon may have benefitted from a higher commission and at the same time did not have to fear a substantial loss of consumers, as the latter may also have faced higher retail prices at competing e-book retailers.

In a recent working paper, Johansen and Vergé (2016) show however that the Retail Price Parity Clauses can have pro-competitive effects if there is competition between e-book sellers and they can sell significant volumes directly to customers. However in the relevant e-book markets, the direct sales channel was of limited importance, and it appears to be unlikely that e-book sellers could credibly threaten to switch a large proportion of their sales to the direct channel.

#### Retail Price Notification Provisions

Because of the commitments that were made binding by the Commission in 2012 and 2013 on the Five Publishers, those Five Publishers could not introduce or maintain any retail price parity clauses for a five-year period in their e-book distribution agreements in the EEA. With those Five Publishers, Amazon replaced the Agency Price Parity Clauses with the Retail Price Notification Provisions.

The Commission found that once notified by an e-book supplier on the basis of those provisions, Amazon typically requested from that e-book supplier that Amazon should be offered the same low retail price (including promotions) that was charged at the competing e-book retailer. The Commission considered that Amazon had a number of measures at its disposal that constituted a credible threat to induce e-book suppliers not to set lower prices on competing e-book retailers’ platforms.^58^ Such measures could be implemented swiftly, without any need of approval by the e-book suppliers and without terminating any agreement, which made them credible threats to induce e-book suppliers to modify their prices on Amazon.

Together with Amazon’s policy of requesting retail price parity, those clauses allowed Amazon to ensure that the retail prices of e-books that were offered on its platform did not exceed the lowest retail prices of the same e-books that were sold via competing e-book retailers with similar effects as already set out for the Retail Price Parity Clauses.

The Commission therefore had the preliminary view that Amazon abused its potentially dominant position by having these provisions included in its e-books distribution agreements.

#### Selection Parity Clauses

The Commission focused on two effects of Selection Parity Clauses: First, the Commission preliminarily found that these clauses are likely to reduce the incentives of e-book suppliers and e-book retailers to develop e-books with innovative features or functionalities such as highly illustrated or enhanced e-books. Second, these clauses are likely to deter their entry and expansion, thereby weakening competition between e-book retailers, to the detriment of consumers.

As regards the first effect, in the absence of Selection Party Clauses e-book suppliers would have had incentives to develop enhanced or highly illustrated e-books, mainly for two reasons. There appeared to be demand for enhanced or highly illustrated e-books of those e-book retailers that use e-book readers or electronic devices on which the illustrations, features, and functionalities of such e-books displayed well. In addition, developing enhanced or highly illustrated e-books with e-book retailers competing with Amazon for their e-book readers or electronic devices could have allowed e-book suppliers to increase the strength of those e-book retailers relative to Amazon and to promote entry and/or expansion.^59^ This could have reduced the e-book sellers’ dependence on Amazon and improve their bargaining position.

However, Amazon’s Selection Parity Clauses either oblige e-book sellers to produce a version of each enhanced or highly illustrated e-book with equivalent features that were compatible with Amazon’s e-book readers or notify Amazon that the e-book may not display well on Amazon’s e-book readers and provide Amazon with all assistance and materials that are reasonably required for Amazon to create an e-book of that title.

Depending on the additional resources that were required to develop equivalent features for Amazon’s e-book readers, this may have significantly increased the development costs in particular for enhanced or highly illustrated titles. The additional costs may have made the development process unprofitable even when taking into account that selling an e-book also on Amazon may give rise to additional revenues. There was evidence that indicated that even the obligation to assist Amazon in the creation of a version that was compatible with Amazon’s e-book readers could have been burdensome (and cause delays), in particular for certain enhanced or highly illustrated titles because it required cost and working time investments from e-book suppliers.

In line with these considerations, there were indications that e-book versions for a number of highly illustrated print books had not been produced in light of the Selection Parity Clauses.

Similarly, e-book suppliers were likely to be induced to keep functionalities of enhanced e-books simple and to avoid interactive and more advanced functions for all e-book retailers in order to simplify the adaption to Amazon’s e-book readers.

Also the ability and incentives of competing e-book retailers to develop innovative e-books and functionalities were likely to be compromised by the Selection Parity Clauses. Competing e-book retailers may have not been able to develop highly illustrated or enhanced e-books to the extent that e-book suppliers were hesitant to cooperate with them in light of the Selection Parity Clauses, even where the e-book reader and formats of those competing e-book retailers display features better than do Amazon’s e-book readers.

Competing e-book retailers may also have invested less in the development of highly illustrated or enhanced e-books as their willingness to invest in the development of such e-books depends on their expected benefits from such investments.^60^

We now turn to the second potential effect of the Selection Parity Clauses: to weaken competition by deterring entry and expansion of e-book retailers. In the absence of Amazon’s Selection Parity Clauses, competing e-book retailers could have been able to offer e-books that were not (or only with less functionality) available on Amazon for the following reasons:

- In light of technical difficulties to convert or to display certain highly illustrated or enhanced e-books on Amazon e-readers, such e-books would likely have been exclusively (or earlier) developed for e-book readers or electronic devices that were supported by competing e-book retailers.
- In light of Amazon’s dominant position in the relevant e-book distribution markets, e-book suppliers may have had an interest in selectively supporting competing e-book retailers: for instance, by exclusive content; special editions (for example with specific features and functionalities); or early release dates.

The presence of Amazon’s Selection Parity Clauses may have limited competing e-book retailers’ scope to differentiate on the basis of content (including by offering titles or special editions not available on Amazon), or of particular features or functionalities of e-books or of earlier release dates.

As a result of Amazon’s Selection Parity Clauses, competing e-book retailers were likely to be hindered from developing and distributing e-books different to those available on Amazon or special editions (including editions with specific features and functionalities), or from choosing early release dates, as the Selection Parity Clauses hindered e-book suppliers from supporting this.

In particular, as set out above, the e-book retailers’ incentives to invest in enhanced or highly illustrated e-books or in non-price related promotional activities were likely to be reduced as a consequence of the Selection Parity Clauses. Lower investments by competing e-book Retailers (either in content or in non-price promotional activities) likely has deteriorated the attractiveness of competing e-book retailers from a customer perspective and hence may weaken competition at the e-book distribution level in the long run.

The risk of reduced competition at the e-book retail distribution level due to Amazon’s Selection Parity Clauses appeared to be more pronounced in markets where Amazon’s position in the retail distribution of e-books was particularly strong. Amazon’s high market share indicated that many e-book customers had a Kindle e-book reader or an Amazon account and that for them it is particularly convenient to buy e-books on Amazon. Buying e-books outside of Amazon’s closed Kindle ecosystem may impose a substantial burden (“switching costs”) on the users of Amazon’s Kindle e-reader. Therefore, to induce potential customers to switch away from Amazon, competing e-book retailers needed to provide additional value to consumers: for example, in the form of differentiated content or early releases of e-books.

Not being able to compete on the basis of a differentiated e-book catalogue or earlier release dates may have not only weakened competing e-book retailers; it could ultimately have forced competing e-book retailers to exit the market or have induced potential competing e-book retailers not to enter the market in the first place. Reduced competition at the e-book retail level may in itself result in higher prices.

#### Business Model Parity Clause

The Commission considered that Amazon’s Business Model Parity Clause is likely to hinder the emergence of alternative business models with regard to the distribution of e-books by: (1) reducing e-book suppliers’ incentives to support, and invest in, new and innovative business models for the e-book supplier’s own platform or for sales channels of e-book retailers; and (2) by reducing the ability and incentives of e-book retailers that competed with Amazon to develop alternative business models. Certain aspects of the assessment of Business Model Parity Clause and of the Selection Parity Clauses are hence conceptually similar.

Regarding the first effect, in relation to e-book suppliers’ incentives, in the absence of a Business Model Parity Clause they appeared to be willing to support alternative business models of competing e-book retailers mainly for two reasons:

- E-book suppliers appeared to have an interest in experimenting with alternative business models—for example with smaller e-book retailers on a smaller scale (one country/region), or on a selection of their catalogue (children books, classics), or targeting only certain customers groups—without having to test such business models on a larger scale for the mass market.
- Supporting alternative business models of competing e-book retailers would have allowed e-book suppliers to increase the strength of those e-book retailers relative to Amazon and to promote entry and/or expansion as set out above.

The presence of the Business Model Parity Clause may have reduced the incentives of e-book suppliers to support and invest in alternative business model in various ways including the following:

- By effectively preventing e-book suppliers from launching alternative business models on their own or with a single or few e-book retailers, the Business Model Parity Clause denied e-book suppliers the opportunity of testing the effect of alternative business models on a small scale. This forced e-book suppliers into a position where they either introduced the alternative business models with Amazon on a larger scale or dropped such projects and did not develop them at all.
- Alternative business models could not be used to strengthen competing e-book retailers relative to Amazon.

In addition, the Business Model Parity Clause may have also negatively affected the e-book retailers’ ability and incentives to develop alternative business models. Specifically, the Commission preliminarily considered that the Business Model Parity Clause diminished the e-book retailer’s incentives to invest in alternative business models in various ways, including the following:

- E-book suppliers became reluctant to support alternative business models as discussed above. This, in turn, undermined the ability of competing e-book retailers to develop such alternative business models as competing e-book retailers need the e-book suppliers’ support, cooperation, and materials in order to implement alternative business models.^61^
- Moreover, even in instances where an alternative business model was launched, the e-book supplier’s obligation to grant the same business model to Amazon was likely to diminish the competing e-book retailer’s incentives to invest in alternative business models for the above-mentioned reasons.

Consequently, in its Preliminary Assessment, the Commission considered that the development of such alternative business models would be reduced or delayed, to the detriment of consumers. Consistent with these considerations, the evidence available to the Commission indicated that the Business Model Parity Clause had prevented the emergence and/or development of alternative models with competitors including: (1) print and e-book bundles; (2) pay-as-you-read and book club models (where readers do not necessarily have to acquire the e-book for an unlimited period of time, but are rather given a license to access only parts thereof); (3) subscription models; and (4) applications for smartphones that give access to e-book versions of classics.

As regards the second effect, Amazon’s Business Model Parity Clause was found likely to reduce the competitiveness of e-book retailers. This was likely to reduce the intensity of competition at the e-book distribution level and was in itself to the detriment of consumers since less competition may result in higher e-book prices and less choice.

A number of observations were important for that conclusion:

- Actual or potential competitors of Amazon were not able to exploit new or alternative business models that are often critical for successful entry and could have been used to challenge Amazon’s market position.
- Not being able to compete on the basis of alternative business models had weakened competing e-book retailers and might have even caused some to exit or not to enter the market.^62^

As already set out above, weaker competition likely in itself results in consumer harm.

### The Commitments

Amazon disagreed with the conclusions of the Commission’s Preliminary Assessment. Nevertheless, it offered the following commitments, which the Commission made binding by issuing the Amazon Decision:^63^

- Not to enforce: (1) a number of parity clauses that required publishers to offer Amazon similar price (including agency price, agency commission, and wholesale price) and non-price terms and conditions (including alternative/new business models, release date and catalogue of e-books, features of e-books) as those offered to Amazon’s competitors or (2) any such clauses that required publishers to inform Amazon about such price and non-price terms and conditions;
- To allow publishers to terminate e-book contracts that contain a Discount Pool Provision upon 120 days’ advance written notice; and
- Not to include, in any new e-book agreement with publishers, any of the clauses that were mentioned above, including Discount Pool Provisions.

### Relation to Investigations in the Online Travel Agency Sector

Since 2010 a number of national competition authorities (“NCAs”) have investigated *inter alia* retail price parity clauses that are used by online travel agencies (“OTA”). Whereas many NCAs expressed concerns about wide retail price MFNs, differing approaches have been implemented concerning narrow MFNs.^64^

In this context, OTAs have argued that narrow parity clauses are indispensable to prevent hotels from free-riding on OTA investments. Their argument is that—absent such parity clauses—consumers will use OTAs to search for and compare hotels, but will then book more cheaply on the hotel’s website, thereby depriving the OTA of commission revenue.

In the OTA cases, the same theories of harm as in the Amazon case for the Retail Price Parity Clauses (see Sect. 3.4.1) were pursued although in those cases a potential infringement of Article 101 of the Treaty on the Functioning of the European Union was investigated. However, given that in many markets several large OTAs operated, the concern that MFNs would soften competition among OTAs appeared to be more pronounced than the concern that weaker OTAs could be excluded. Prima facie, it seems indeed plausible that potential exclusionary effects of retail price MFNs are more pronounced in environments where: (1) very strong firms are competing against relatively small/weak competitors; (2) the incumbent enjoys a quality advantage; or (3) the market position of the incumbents is protected by significant switching costs.

Second, the direct sales channel played a much more prominent role in the OTA investigations compared to the Amazon case. Indeed, in the relevant e-book markets the direct channel appeared to play a rather marginal role. This probably has to do with the benefits that are linked to using a given platform (for example, the Kindle devices work with e-books in the proprietary Amazon format). Consequently, also the claim that parity clauses are needed to restrict free-riding appears to be less convincing in the Amazon case.

## Conclusion

The 2016–2017 season was quite interesting, in particular with the emphasis—in mergers—on theories of harm based on innovation. On the antitrust side, MFN clauses were analysed not only in the Amazon case, but also in the context of the investigations in the OTA sector. Given the current strong merger pipeline, the Google cases and the European Court of Justice judgement in the Intel case,^65^ the year to come promises to be busy for the DG Comp and the CET on all fronts.

## Footnotes

1 Case AT.39850. See DG Comp’s press release available at http://europa.eu/rapid/press-release_IP-16-2446_en.htm as well as DG Comp’s article of last year (Claici et al. 2016).

2 Case AT.39745. See DG Comp’s press release available at http://europa.eu/rapid/press-release_IP-16-2586_en.htm.

3 Case AT.40023. See DG Comp’s press release available at http://europa.eu/rapid/press-release_IP-16-2645_en.htm.

4 Case AT.39759. See DG Comp’s press release available at http://europa.eu/rapid/press-release_IP-16-3116_en.htm.

5 Case AT.40153. See DG Comp’s press release available at http://europa.eu/rapid/press-release_IP-17-1223_en.htm.

6 Case AT.39740. See DG Comp’s press release available at http://europa.eu/rapid/press-release_MEMO-17-1785_en.htm.

7 Detailed statistics on the number of merger notifications and Decisions are available at http://ec.europa.eu/competition/mergers/statistics.pdf.

8 Details on the European Union merger regulation are available at http://ec.europa.eu/competition/mergers/procedures_en.html.

9 The transactions that have been prohibited between January 2016 and July 2017 are: M.7612—Hutchison 3G UK/Telefonica UK (May 2016); M.7995-Deutsche Börse/London Stock Exchange Group (March 2017); and M.7878—HeidelbergCement/Schwenk/Cemex Hungary/Cemex Croatia (April 2017). The notification of the case M.7477—Halliburton/Baker Hughes was withdrawn in May 2016 after the issuance of a Statement of Objections.

10 A similar analysis of innovation competition in an antitrust context can also be found in paragraph 26 of the European Commission Guidelines on Technology Transfer Agreements, and in paragraphs 199-122 of the European Commission Horizontal Cooperation Guidelines.

11 For a more detailed review of the main benefits of competition for innovation, the legal framework for analysing innovation effects, and a more detailed review of recent cases, see Competition policy brief “EU merger control and innovation”, dated April 2016, available at http://ec.europa.eu/competition/publications/cpn/.

12 M.7326—Medtronic/Covidien (November 2014); M.7275—Novartis/GSK oncology business (January 2015); M.7559—Pfizer/Hospira (August 215); M.7278—GE/Alstom (September 2015); M.7477 – Halliburton/Baker Hughes (withdrawn in May 2016); M.7995—Deutsche Börse/London Stock Exchange Group (March 2017); and M.7932—Dow/DuPont (March 2017).

13 Case T-175/12—*Deutsche Börse AG v. European Commission* (March 2015), following the prohibition of M.6166—Deutsche Börse/NYSE Euronext (February 2012), available at http://curia.europa.eu/juris/liste.jsf?language=en&num=T-175/12.

14 The R&D in the pharmaceutical industry and in the agrochemical industry are characterised by long and costly R&D processes that are organised through standardised phases (for example, the three-phase development system for drugs in clinical development). The Commission assessed overlaps between pipelines in advanced stage of development and current products (in Medtronic/Covidien and Pfizer/Hospira), overlaps between pipelines in all phases of development (in Novartis/GSK oncology), and overlaps between pipelines and products, between pipelines, and between lines of research (in Dow/DuPont—see below).

15 This assessment was made in GE/Alstom and in Halliburton/Honeywell.

16 Statistics available at http://ec.europa.eu/competition/publications/annual_report/2016/part2_en.pdf

17 Case SA. 38373. SeeDG Comp’s press release available at http://europa.eu/rapid/press-release_IP-16-2923_en.htm.

18 Case SA. 44888. See DG Comp’s press release available at http://europa.eu/rapid/press-release_IP-16-3085_en.htm.

19 See http://ec.europa.eu/competition/state_aid/modernisation/state_aid_evaluation_methodology_en.pdf.

20 For a discussion of the capacity mechanisms sector enquiry, see Claici et al. (2016).

21 A (provisional) non confidential version of the decision is available at http://ec.europa.eu/competition/elojade/isef/case_details.cfm?proc_code=2_M_7932. See also Bertuzzi et al. (2017) for a comprehensive summary of the case.

22 Dow is a US-based chemicals company that is active in plastics and chemicals, agricultural sciences, and hydrocarbon and energy products and services. DuPont is also a US-based chemicals company that produces a variety of chemical products, polymers, agro-chemicals, seeds, food ingredients, and other materials. Innovation concerns were identified in relation to crop protection.

23 Forthcoming products refer in this specific case to pipeline products at the development stage.

24 A line of research essentially refers to a particular pipeline product at the discovery stage (with the corresponding scientists, patents, assets, equipment).

25 See for example Baker (2007), and Shapiro (2012). These papers also point to a number of empirical studies that support the finding that competition tends to favour innovation.

26 See D’Aspremont and Jacquemin (1988), Lopez and Vives (2016).

27 In the literature, however, this concept has broader connotations (e.g. it is used with reference to changes in product differentiation or collusion) that do not always correspond to the loss of product market competition due to a merger.

28 However, even if the effects of a merger on future product market competition were to increase innovation incentives and be so strong as to neutralise the adverse impact on innovation due to a loss of innovation competition, consumers would still be harmed by a significant loss of future product competition due to the merger.

29 See Motta and Tarantino (2017) and Lopez and Vives (2016). In addition, Federico et al. (2017a, b) show that, even when using a demand model such that the loss of product competition tends to increase innovation incentives, this effect is not sufficiently strong to compensate for the loss of innovation incentives due to the innovation rivalry channel. Chen and Schwartz (2013), in contrast, find that a merger may increase innovation in a Hotelling model with two products. In the model, however, innovation can take place only in one product and that downplays the importance of innovation diversion.

30 See, e.g., Gilbert (2006).

31 Following successive waves of consolidation, the number of R&D-integrated companies has decreased from more than 40 firms in 1960-1980 to only five companies.

32 The discovery process takes 3-to-4 years and includes the synthesis of candidate molecules (chemical activity), which are then screened to determine the biological activity of the molecule. Molecules are moved forward in the discovery process based notably on their efficacy, toxicological and environmental properties, and fit with company targets.

33 The development process takes 5-to-6 years. Regulatory studies are a large part of the development efforts. Field trials represent the largest single cost in the development cycle. After registration is obtained, a company would typically start with some initial preparations/mixtures to start the roll-out of the AI, with later extensions to new mixtures, new uses, and new countries.

34 In its assessment, the Commission considered several elements to assess closeness of innovation competition, such as: the crops and pests that are targeted by the molecules under development; the mode of actions (i.e., how the molecules affect the pests’ targeted systems); the chemical classes; the benchmarking with competitors’ molecules; and the monitoring of research programs of competitors.

35 An argument that was raised in this case is that any competitive overlaps between a discovery pipeline of one merging party and an existing product of the other merging party would not lead to any cannibalisation concern since biological resistance (existing products would become obsolete once the targeted pests develop resistance to them) and regulation (existing products having a risk of being restricted or banned from the market for toxicity reasons) would limit the future profits that would be expected from existing products. However, the Commission found that existing products from the merging parties were expected to maintain relatively high sales and margins at times when the other merging parties’ pipelines would have reached the market, therefore confirming that cannibalization of existing products would matter.

36 An issue that was raised in this case concerns the uncertainty that characterises pipelines at the discovery stage. While pipelines at the discovery stages have an uncertain outcome, the Commission considered that one should not confuse the uncertain outcome of innovation with whether or not competition concerns are present. Even if there is uncertainty as to the outcome of the innovation process, a merger between firms with competing lines of research is likely to affect the incentives to invest in research negatively. Therefore, while the outcome of any given innovation effort may be uncertain, this does not mean that competition concerns in relation to innovation efforts are not warranted.

37 Several Japanese discoveries were also further developed by the five global integrated players, suggesting that even the discovery capabilities of Japanese companies have some limitations. In that case, Japanese companies owned the initial discovery patents, but the subsequent discovery patents were owned by one of the five global R&D companies. While the patents of Japanese companies would get some citations, their underlying discoveries would not, in such instances, be helpful to bring a product to the European market.

38 Excluding the Japanese companies is technically equivalent to reallocate their patents’ shares proportionally to each of the five global R&D integrated companies. However, the Commission found that among the products that were based on AIs that were co-developed by Japanese companies and by one the five global R&D-integrated companies, the merging parties accounted for the vast majority of the associated sales; this therefore suggested that the majority of patent shares of Japanese companies should have been reallocated to the merging parties. In that sense, excluding the Japanese companies can be considered as a conservative approach.

39 See Tratjenberg (1990), Griliches (1990), Jaffe et al. (1993), Aghion et al. (2005), Hall et al. (2005), Cohen (2010), Ernst and Omland (2011), and Bloom et al. (2013).

40 PatentSight is a service company that was used by the Parties. See https://www.patentsight.com/. See also Ernst and Omland (2011) for a description of the methodology that is used by PatentSight.

41 To protect an innovation, patent applicants seek protection in several countries so that, typically, there is more than one patent publication per innovation. A patent family groups and identifies all patents that describe the same innovation. PatentSight allows a focus on patent families by avoiding counting multiple patents that are related to the same innovation.

42 The PatentSight’s web-interface also provided a measure of patent quality, which is proprietary to PatentSight. In its assessment, the Commission used in particular a measure called “Technology Relevance”, which is based on the number of worldwide citations that are received from later patents: adjusted for age, patent office practices, and technology fields.

43 See Annex 1 of the Dow/DuPont Decision for further details.

44 See Hall et al. (2005). In their study, they find that for firms having an average-size patent portfolio (about 200 patents in their study), the effect of internal citations on firm’s value is important, it is even more important for firms with smaller portfolios, but for firms with an important portfolio (about 1000 patents in their study), internal citations have no impact on firm’s value and above 1000 patents the impact of internal citations on firm’s value is even negative.

45 Based on total citations, one merging party had an average patent quality for its patents that was 2.5 times higher than its competitors, while based on external citations it had an average quality for its patents that was more than four times higher.

46 The Commission also checked that its conclusions were robust when total citations (i.e., including internal citations) were used to measure patent quality. The main change in the results was the ranking of one merging party, which was the number one innovator in crop protection when external citations were used, and the number two innovator in crop protection when total citations were used.

47 Note that the percentiles have been defined by reference to the ranking of the quality of all competitors’ patents together.

48 Another sample of patents (the top 10%) was also considered: that is, patents above the 90^th^ percentile in terms of quality. The Commission found that this sample of patents included some innovations that were related to the so-called “blockbuster” products: the most successful products in term of sales.

49 Hall et al. (2005) show that for firms with fewer than the median number of citations per patents, it makes no difference how far below the median they fall (which includes as well patents with zero citations), while firms with more than the median number of citations per patent exhibit a very significant increase in market value. These findings suggest that patents with quality below the median quality do not bring significant value to firms. See also Coad and Rao (2008), who find that innovativeness appears to have a small or no influence on firm growth for the median firm.

50 Tratjenberg (1990) finds that the value of an innovation for customers is more skewed than what could be inferred from a count of citations, and therefore that a non-linear weight should be applied to citations to measure the value of an innovation. See also Harhoff et al. (2003), who study the value of inventions by using estimates that are obtained directly from patent holders through a survey. They find that the distribution of patented innovation values is highly skewed, and that for the top quality patents their estimated value from surveyed customers is significantly larger than are other estimates from the literature that uses metrics that are based on patent data.

51 The Commission also found a significant number of cross-citations between some patents of the merging parties (i.e. the merging parties were citing each other to a significant extent). The review of the internal documents of the parties indicated that the research projects that are related to these specific patents had in common some important technical characteristics, such as similar chemical classes or modes of actions, which indicated a very significant degree of innovation closeness.

52 See the press release available at http://europa.eu/rapid/press-release_IP-17-2182_en.htm.

53 More information, including the Decision of 4 May 2017 in case AT.40153 E-book MFNs and related matters, is available on the Commission’s competition webpage: http://ec.europa.eu/competition/elojade/isef/case_details.cfm?proc_code=1_40153.http://ec.europa.eu/competition/antitrust/cases/dec_docs/40153/40153_4392_3.pdf.

54 See Commission Decision of 12 December 2012 in Case No COMP/39847—E-Books and Commission Decision of 25 July 2013 in Case No. COMP/39.847—E-Books.

55 For simplicity, this definition also includes promotions and discounts. In the Amazon Decision, the Commission distinguishes between the Agency Price Parity Clause and the so-called Promotion Parity Clause. The latter clause obliges the e-book supplier to offer to Amazon any promotional agency price or promotional content that the e-book supplier offers through an e-book retailer other than Amazon.

56 Since the Commission ended its investigation by a commitments decision, only a preliminary assessment was carried out.

57 See Decision of 4 May 2017 in case AT.40153 E-book MFNs and related matters, Sect. 5.3.4.3. for further details on the Discount Pool Provision and on the Commission’s assessment of the possibility to replicate the effect of an Agency Price Parity Clause.

58 Amazon could for example remove the buy button for one or several e-books on its platform (as it had done previously), exclude e-books of a publisher from all promotional activity, remove the pre-order buttons or prominently display banners that attempt to dissuade potential buyers by referring to seemingly more attractive alternative e-books.

59 Increased sales of competing e-book retailers are likely to increase their competitiveness: Once customers acquire a certain e-book reader or get used to the apps of a given competing e-book retailer, it becomes more convenient for those customers to buy further e-books from that e-book retailer. In other words, Amazon customers that are already used to competing e-book retailers have lower switching costs when buying additional e-books from those competing e-book retailers and are therefore more likely to consider buying again from those competing e-book retailers.

60 If e-book suppliers are obliged, due to the Selection Parity Clauses, not to differentiate their e-books offerings across e-book retailers, competing e-book retailers may anticipate that many customers will buy a newly developed e-book on Amazon in light of Amazon’s ecosystem and its high market share. This in turn significantly reduce the anticipated revenues of competing e-book retailers.

61 For example, in order to implement a subscription business model, competing e-book retailers need to be authorised by the e-book suppliers to provide access to the e-books to consumers on a subscription basis.

62 There were indications that a number of companies—notably smaller ones and start-ups—could not enter the e-books distribution markets or faced difficulties in developing their e-book distribution business because of the inability and unwillingness of e-book suppliers to license their catalogue to them for alternative business models.

63 The commitments are available at http://ec.europa.eu/competition/antitrust/cases/dec_docs/40153/40153_4393_3.pdf.

64 For an overview, see, e.g., European Commission (2017), p. 4. Whereas wide MFNs provide that the price that is quoted on a retailer's platform must not exceed the prices that are available on other sales channels (including competing retailer's platforms), narrow MFNs provide that the price that is on a given retailer's platform must not exceed the price for direct sales.

65 Case C-413/14 P Intel Corporation Inc. v Commission. See the Court of Justice’s press release available at https://curia.europa.eu/jcms/upload/docs/application/pdf/2017-09/cp170090en.pdf.
